# Relationship between hepcidin and oxidant/antioxidant status in calves with suspected neonatal septicemia

**DOI:** 10.14202/vetworld.2016.1238-1241

**Published:** 2016-11-14

**Authors:** E. E. Erkilic, H. M. Erdogan, M. Ogun, A. H. Kirmizigul, E. Gokce, M. Kuru, A. Kukurt

**Affiliations:** 1 Department of Internal Medicine, Faculty of Veterinary Medicine, University of Kafkas, 36100, Kars, Turkey; 2 Department of Biochemistry, Faculty of Veterinary Medicine, University of Kafkas, 36100, Kars, Turkey; 3 Department of Obstetrics and Gynecology, Faculty of Veterinary Medicine, University of Kafkas, 36100, Kars, Turkey

**Keywords:** Fe, hepcidin, oxidative stress, septicemia

## Abstract

**Aim:**

This study has been conducted for the purpose of determining serum hepcidin, total antioxidant status (TAS), total oxidant status (TOS), and Fe levels in calves with suspected neonatal septicemia before and after treatment and the clinical significance of hepcidin in calves with suspected neonatal septicemia.

**Materials and Methods:**

The study material consisted of 15 calves of different ages and sexes brought to the Training, Research and Application Center at the Kafkas University Faculty of Veterinary Medicine with suspected neonatal septicemia. 8.5 mL of blood was drawn from the jugular vein of each animal into coagulant tubes before and after treatment for one-off biochemical analyses and centrifuged. After this, the serum was separated. Hepcidin, TAS, TOS, and Fe levels in the serum were measured.

**Results:**

While pre-treatment hepcidin levels were 58.42±3.46 ng/mL, post-treatment levels were 46.87±2.98 ng/mL (p<0.05). Pre-treatment Fe levels were 60.13±7.27 µg/dl, while post-treatment levels were 83.1±8.09 µg/dl (p<0.05). The changes in the TAS and TOS levels were also found to be statistically significant.

**Conclusion:**

In light of the fact that hepcidin plays a role function in the regulation of Fe as well as the fact that Fe is a significant nutritional source for many microorganisms, it was concluded that hepcidin may play a significant role in nutritional immunity and the pathogenesis of diseases.

## Introduction

Neonatal calf septicemia causes high morbidity and mortality and is one of the leading and most significant difficulties in raising cattle. Calf septicemia is the main cause of death in the neonatal period [1]. Its etiology involves bacteria (commonly *Escherichia coli*), viruses (rota and coronavirus), parasites, and other factors. As the disease progresses quickly and is lethal, diagnosis and treatment should be initiated as quickly as possible [2].

Hepcidin is a low molecular weight, antimicrobial peptide hormone and was first discovered in human urine [3]. It is produced by the liver as a first-line response to inflammatory reactions and high Fe concentrations [4,5]. Hepcidin plays a fundamental role in the regulation of Fe metabolism [6], which is a part of foundational cellular functions and thus of vital importance. On the other hand, by participating in redox reactions leading to the production of reactive oxygen species (ROSs), Fe also causes oxidative stress. Therefore, Fe has been regarded as a potentially toxic element to cells [7]. Fe also plays an important role in pathogenesis of bacterial infections as bacteria utilize Fe for survival, growth and proliferation; therefore, it is of paramount importance to control the Fe metabolism [6]. It is well known that the abundance of Fe suppresses defense system leading host vulnerable to infections. There is a significant relationship between Hepcidin, Fe metabolism, inflammation, and the immune system. The fact that hepcidin plays an active role in the regulation of Fe release from macrophages and in the control of excessive Fe absorption from the duodenum is well documented [6]. Hepcidin is a part of the natural defense mechanism, thus it limits the amount of Fe that can be utilized by pathogens [8]. In inflammatory conditions, hypoferremia is an important first-line protective mechanism in response to infections [9]. Fe also participates in redox reactions, causing the production of ROS, and thus leading to oxidative stress [7]. Free radicals play a significant role in the pathogenesis of many diseases [10]. Newborns are subject to oxidative stress during birth. It is also reported that in livestock diseases, especially enteritis and pneumonia, antioxidant capacity is efficacious [11].

This study was designed to determine the clinical significance of hepcidin in calves with suspected neonatal septicemia by evaluating serum hepcidin, total antioxidant status (TAS), total oxidant status (TOS), and Fe levels in calves suspected of neonatal septicemia before and after treatment.

## Materials and Methods

### Ethical approval

This study was conducted after obtaining approval from the Mehmet Akif Ersoy University Animal Experiments Local Ethics Committee (MAKU-HADYEK-Submission: 2014/77).

### Animals

The study consisted of 15 calves with suspected neonatal septicemia aged between 1 and 10 days old admitted to the Teaching Hospital of Veterinary Medicine. Suspected septicemia was diagnosed based on clinical (diarrhea, weakness in or absence of sucking reflex, the calf being in a supine position on the ground or being unable to stand, severe dehydration, abnormal rectal temperature [hypo- or hyperthermia], mucosal hyperemia, and full sclera) and hematological (increase in white blood cell [WBC] count) examinations; the animals were suspected to have septicemia [12,13]. The animals were given standard treatment (antibiotic, nonsteroidal anti-inflammatory drugs, vitamin C, fluid therapy, and intestinal astringent). For determination of serum hepcidin, TAS, TOS, Fe levels, and hematological parameters; blood samples were taken before and after treatment in all cases. 8.5 mL of blood was taken from the jugular vein of each animal into coagulant tubes for biochemical analysis, and 3 mL blood was taken into ETDA tubes for hematological analysis. Samples were centrifuged at 3000 rpm for 10 min, and the serum was harvested and kept at −20°C until the analysis. Serum hepcidin (Mybiosource^®^), TAS (Rel Assay Diagnostics^®^), and TOS (Rel Assay Diagnostics^®^) were determined using commercial ELISA kits, and Fe value was measured spectrophotometrically. Hematological (WBC, lymphocyte [LYM], red blood cells [RBC], mean corpuscular volume (MCV), and hematocrit [HCT]) analysis was performed on blood counter (VG-MS4e^®^, Melet Schloesıng, France).

### Statistical analysis

The results were evaluated using the t-test in the SPSS^®^ (SPSS 20, USA) statistical package program to determine the differences between values before and after treatment.

## Results

### Clinical examination findings

Calves with suspected septicemia exhibited clinical signs of loss of appetite, fatigue, indifference to surroundings, reduced/absence of sucking reflex, cool extremities, inability to stand, diarrhea, eye sinking into their sockets, and hyperemia in the conjunctiva. The average body temperature, heart rate, and respiratory rates of the animals were 37.18±0.13°C, 104±4.33/min, and 28.86±0.75/min pre-treatment; and 38.54±0.1°C, 107.53±2.20/min and 26.40±0.36/min post-treatment, respectively.

### Biochemical findings

The changes in hepcidin, TAS, TOS and Fe levels in the calves with suspected septicemia before and after treatment are given in Table-1. After treatment, serum hepcidin and TOS levels were significantly lower than before treatment in calves. On contrary, serum TAS and Fe levels were significantly higher than before treatment (Table-1).

**Table 1:** Serum biochemical changes (X̅±SE) in calves before and after treatment (n=15).

Parameters	Before treatment	After treatment	p
Hepcidin ng/mL	58.42±3.46	46.87±2.98	p<0.05
TAS mmol Trolox equivalent/L	0.50±0.02	0.58±0.03	p<0.05
TOS mM H_2_O_2_ equivalent/L	5.30±0.74	2.13±0.2	p<0.05
Fe µg/dL	60.13±7.27	83.1±8.09	p<0.05

SE=Standard error, TAS=Total antioxidant status, TOS=Total oxidant status

### Hematological findings

The treatment of calves resulted in significant changes in the hematological parameters that were examined except for RBC. The WBC count, LYM count, MCV and HCT significantly changed after treatment when compared to values obtained before treatment (Table-2).

**Table 2:** Hematological changes (X̅±SE) in calves before and after treatment (n=15).

Parameters	Before treatment	After treatment	p value
WBC (10^9^/L)	16.30±1.25	11.47±0.79	p<0.01
LYM (10^9^/L)	6.05±0.83	3.5±0.38	p<0.01
RBC (10^12^/L)	9.07±0.42	11.01±0.84	p>0.05
MCV (fl)	44.57±1.13	41.25±0.96	p<0.05
HCT (%)	50.64±2.7	40.99±10.57	p<0.05

WBC=White blood cell, RBC=Red blood cells, MCV=Mean corpuscular volume, HCT=Hematocrit, LYM=Lymphocyte, SE=Standard error

## Discussion

This study aimed to determine the clinical importance or use of hepcidin by comparing the values of serum hepcidin, TAS, TOS and Fe levels in calves with suspected neonatal septicemia before and after treatment.

Clinicians rely on clinical and laboratory examinations of patients to form a working diagnosis, so hematological and serum biochemical parameters are usually used for this purpose [14]. The hematological parameters (WBC, HCT, LYM, and MCV) evaluated in this study were comparable with those reported by others in neonatal calves with diarrhea and suspected septicemia [15-17]. Treatment significantly corrected to normal values the hematological parameters that were examined with the exception of RBC. Pre-treatment leukocyte count was high because of the inflammation that occurred in the organism, and that the HCT levels were high due to the dehydration that occurred due to diarrhea.

Hepcidin is controlled by the presence of inflammation in the body, Fe storage, and erythropoietic activity in the bone marrow and plays a primary role in the homeostasis of Fe [4]. The increase in tissue and plasma Fe levels stimulates the synthesis of hepcidin and reduces Fe release and enteric Fe absorption from macrophages and hepatocytes [18]. Increased hepcidin concentrations during inflammation and infection reduce serum Fe levels by decreasing Fe release from macrophages and hepatocytes, and thus Fe required for microorganisms and tumor cells is restricted [19].

Serum hepcidin levels in calves with suspected septicemia were significantly high before treatment when compared to after treatment; also Fe levels were lower before treatment when compared to after treatment in this study. This situation could be related to the interaction between hepcidin and Fe and also gives credence to the role of hepcidin in the hemostasis of Fe during inflammation and infection. As in our study, Fe levels are well known to decrease in diarrheic calves when compared to healthy calves [20,21]. Although no study exists reporting hepcidin concentration in diseased calves, studies in human subjects show that cord blood hepcidin levels might be an important indicator in diagnosing early-onset of neonatal sepsis. The cord blood hepcidin levels of neonatal infants with sepsis varied between 118.1 and 8400 ng/mL and were significantly higher than the healthy infants [22]. A similar result was reported that hepcidin concentrations in neonatal infants with sepsis were significantly higher than in healthy infants [23]. These findings along with our results add credence to the idea that hepcidin-Fe interaction may play a role in the pathogenesis of septicemia.

The production of free oxygen species causes alterations in protein, lipid, and DNA during oxidative stress and leads to the development of lesions in the organs [24]. Free iron has toxic characteristics as it catalyses the production of ROSs [25] and thus causes oxidative stress [26]. The role of Fe in the development of oxidative stress may once more show the importance of hepcidin, as an important Fe regulator, with regard to enhancing antioxidant capacity through inhibiting utilization of Fe by the organism as well as the host cells.

The antioxidant and oxidative system are in a constant state of balance in the organism. Any event breaking up this balance in favor of the oxidative stress molecules will cause cell damage [27,28]. The host cells initiate the antioxidant system in case of exposure to oxidative stress [27]. Kabu *et al*. [16] reported TOS and TAS values in neonatal calves with diarrhea as 13.47±0.81 µmol H_2_O_2_/L and 0.51±0.02 mmol Trolox-equivalent/L, respectively, and treatment of these calves caused changes in these values of 11.21±0.26 µmol H_2_O_2_/L and 0.55±0.02 mmol Trolox-equivalent/L, respectively. Studies also reported that parameters used for oxidative stress (malondialdehyde) were higher [29] and antioxidant parameters (superoxide dismutase [21], TAS) were lower in diarrheic calves [29]. Similarly, in our study, TAS level was significantly lower and TOS level was significantly higher in diarrheic calves before treatment, and treatment caused corrections in these parameters. Decrease in TAS and increase in TOS levels demonstrated that oxidative stress was evident in the diseased calves in our study. Increased TOS and hepcidin levels before treatment are thought that associated with inflammation. After treatment increased TAS and decreased hepcidin levels support this opinion.

## Conclusion

Hepcidin may play an important part in non-specific immunity and is a key molecule that plays a role in the pathogenesis of diseases by enhancing the development of antioxidant system. However, more detailed studies are needed on the role of hepcidin in the pathogenesis of septicemia.

## Authors’ Contributions

This work was carried out in collaboration between all authors. EEE, HME and AHK: Designed the experimental procedures. EEE, EG and MK: Conducted the research work. EEE, AHK, MO and AK: Helped in laboratory analysis. All authors read and approved the final manuscript.
